# Psychiatric difficulties in females with fragile X syndrome: a systematic review and meta-analyses

**DOI:** 10.3389/fpsyt.2026.1886787

**Published:** 2026-07-08

**Authors:** Lauren Jenner, Christina Koenig, Rachel M. Hantman, Abigail L. Hogan, Jane E. Roberts, Jessica Klusek

**Affiliations:** 1 Department of Communication Sciences and Disorders, Arnold School of Public Health, University of South Carolina, Columbia, SC, United States; 2 Department of Psychology, University of South Carolina, Columbia, SC, United States; 3 Carolina Autism and Neurodevelopment Research Center, University of South Carolina, Columbia, SC, United States

**Keywords:** autism, females, fragile X syndrome, mental health, meta-analysis, sex differences

## Abstract

**Introduction:**

Fragile X syndrome (FXS) is the most common monogenic cause of autism, yet research has largely prioritized males. Females show more variable phenotypes and remain comparatively understudied. This paper provides the first systematic review and meta-analysis of psychiatric difficulties in females with FXS, examines associations with intellectual ability and co-occurring autism, and evaluates sex representation within the FXS psychiatric literature.

**Methods:**

Systematic searches of PsycINFO, MEDLINE, Embase, and Web of Science identified English-language, peer-reviewed studies published between 1980 and 2025 reporting psychiatric conditions or symptoms in females with FXS. Of 3,533 records screened, 37 studies met inclusion criteria. Findings were synthesized narratively, and random-effects meta-analyses estimated prevalence of psychiatric difficulties.

**Results:**

A pronounced sex imbalance was observed, with male-focused studies outnumbering female-focused studies by approximately 4:1. Anxiety, depression, attention-deficit/hyperactivity disorder (ADHD), aggression, and self-injury were most commonly reported. Meta-analyses indicated high prevalence of anxiety (57%) and depression (41%), with substantial heterogeneity partly attributable to age variability across samples. Less heterogeneous prevalence estimates were observed for ADHD (33%), aggression (20%), and self-injury (14%). Co-occurring autism was consistently associated with greater psychiatric vulnerability, whereas intellectual ability showed no consistent associations.

**Conclusion:**

Females with FXS exhibit a marked vulnerability to psychiatric difficulties. However, their ongoing underrepresentation in research suggests that the present findings likely underestimate the true level of psychiatric risk. These findings highlight the importance of developing more refined, sex-specific models of psychopathology in FXS, informed by advances in autism research, to enhance the recognition, understanding, and support for females.

## 1 Introduction

Fragile X syndrome (FXS) arises from an expansion exceeding 200 CGG trinucleotide repeats in the *Fragile X Messenger Ribonucleoprotein 1* (*FMR1*) gene located on the X chromosome. This expansion results in epigenetic silencing of *FMR1*, thereby preventing the production of Fragile X Messenger Ribonucleoprotein (FMRP) – a protein essential for brain development and synaptic plasticity (1–3). Clinically, FXS affects approximately 1 in 2,500 to 7,000 males and 1 in 2,500 to 11,000 females (4, 5). Its neurobehavioral phenotype is characterized by intellectual disability (ID) (6), co-occurring autism (7), and autism-like traits (8). Psychiatric difficulties are also prevalent, including anxiety (9), attention-deficit/hyperactivity disorder (ADHD) (10), and self-injurious and aggressive behaviors (11, 12).

Despite the seemingly well-defined clinical picture, much of the available evidence has been derived predominantly from male samples. This has produced a male-centric understanding of FXS and corresponding clinical guidance that may not fully reflect the female phenotype. Consequently, the more variable and less predictable presentation in females remains underexplored and underserved. To address this critical gap, the current systematic review and meta-analysis focus on psychiatric difficulties in females with FXS, examining their associations with intellectual ability and autism. It also provides the first systematic evaluation of sex representation across research on psychiatric difficulties in FXS over recent decades.

### 1.1 Female phenotypic variability and research barriers in FXS

The limited understanding of the female FXS phenotype largely stems from the substantial variability observed among affected females. The presence of a second, typically unaffected X chromosome provides partial protection, as some cells continue to express *FMR1* and produce FMRP. The degree of this protective effect depends on several interacting biological factors, including CGG repeat size mosaicism (inherent to all females), methylation of the expanded allele, and the activation ratio – the proportion of cells in which the affected X chromosome is active (13–15). These mechanisms contribute to a phenotype in females that is markedly more heterogeneous and less predictable than in males (14). As a result, females with FXS exhibit a wide range of intellectual abilities – from moderate or severe ID to above-average intelligence (16). This variability has perpetuated the misconception that FXS is uniformly “milder” in females (17) and has even fostered the notion of a “fragile X spectrum”, with males at one end and “unaffected” females at the other (18). Consequently, research and clinical efforts have historically focused on males, presumed to have greater support needs, while neglecting the complex and variable presentation in females.

Methodological challenges have further perpetuated this bias. Few psychiatric assessment tools are validated across the full range of intellectual abilities seen in FXS, limiting accurate characterization of female presentations. Instruments such as the Anxiety, Depression, and Mood Scale (ADAMS; 19) and the Aberrant Behavior Checklist (ABC; 20) were developed for individuals with ID, whereas more mainstream measures like the Child Behavior Checklist (CBCL; 21) may not retain validity in this population (22). Consequently, comparisons between females and males with FXS are often confounded, as group-level analyses may inadvertently contrast individuals with ID against those without. Although overlap exists – some females present with severe ID and some males fall within the borderline range (23) – concern remains that apparent sex differences are often artifacts of sampling or measurement bias.

Moreover, the greater variability observed among females necessitates larger sample sizes to achieve adequate statistical power. However, given the rarity of FXS, achieving such samples remains a major challenge. Although rarity is an inherent limitation in genetic syndrome research (24), it presents particular difficulties when studying females with FXS, who exhibit a lower clinical prevalence than males (4, 5). This apparent disparity likely reflects diagnostic bias, as females without intellectual disability often go undiagnosed — perpetuating under recognition and their limited representation in research. Consequently, methodological and sampling constraints have produced a research base disproportionately focused on males — valuable, yet incomplete in representing the full phenotypic spectrum.

Importantly, these challenges extend beyond individual studies to highlight a broader systemic issue within research funding. Studies focused on females are likely perceived as less feasible due to methodological constraints and are therefore less likely to secure grant support. Consistent with this pattern, data from the National Institutes of Health RePORTER database (searches for “fragile X” or “*FMR1*”, accessed December 2025) indicate that research attention to females with FXS has been far more limited than to males. This creates a self-perpetuating cycle in which funding priorities reinforce existing research gaps, continuing to hinder progress in understanding and addressing the distinct needs of females with FXS.

### 1.2 The relevance of psychiatric difficulties to female outcomes

The resulting knowledge gap carries tangible consequences. Females with FXS are frequently underdiagnosed, often identified only after a male relative receives a diagnosis (25). Even when diagnosed, genetic counseling has historically centered on male presentations, providing limited information about the distinct challenges faced by females and often relying on the outdated notion that their symptoms are “milder” (26). Although more recent clinical guidelines acknowledge psychiatric and emotional difficulties as key concerns for females (27), these recommendations remain general and lack specificity regarding symptom onset, prevalence, or developmental trajectory.

This is particularly concerning given that, despite often higher intellectual abilities, many females with FXS do not attain greater functional independence than males. National survey data (28) indicate that a large proportion of females require assistance in daily living (63%) and continue residing with parents well into adulthood (51%), with affect problems (i.e., anxiety/depression) and social challenges emerging as the strongest predictors of reduced independence. In contrast, for males, limitations in adult independence are more closely linked to deficits in adaptive functioning, reflecting the central role of co-occurring intellectual disability in the male phenotype. This divergence underscores a critical issue: intellectual ability continues to serve as the default proxy for clinical need in FXS. To more accurately represent and support the experiences of females, research must move beyond intellectual functioning as the primary indicator of vulnerability and instead prioritize psychiatric difficulties – particularly anxiety and depression – which appear to be key determinants of independence, well-being, and long-term quality of life.

To date, only one systematic review and meta-analysis has focused exclusively on females with FXS. Marlborough et al. (29) reported an elevated prevalence of autism among females with FXS (14%) compared to the general population (2%; 30), aligning with the recognition of FXS as the most common monogenic cause of autism. However, it is important to emphasize that neurodivergence itself is not inherently pathological. Evidence from broader neurodivergent female populations shows that the most debilitating challenges often stem from co-occurring psychiatric difficulties such as anxiety, depression, and suicidality (31–34);. These difficulties likely arise from navigating predominantly neurotypical environments and receiving inadequate or poorly tailored support (35, 36). Although this framework has not yet been explicitly applied to FXS, existing data (28) suggest similar vulnerabilities among females with FXS. Emerging evidence indicates growing research interest in recent years. However, existing findings remain fragmented and unsynthesized, limiting translation into meaningful clinical guidance for families and practitioners. Together, these gaps underscore the urgency and relevance of the present review.

### 1.3 The present review

This review represents the first systematic synthesis of the literature specifically examining psychiatric difficulties in females with FXS. Where data permit, meta-analyses estimate the prevalence of key difficulties, including anxiety, depression, ADHD, aggression, and self-injury. The review further explores associations with intellectual ability and co-occurring autism to identify factors that may increase vulnerability to psychiatric symptoms. Additionally, it evaluates patterns of sex representation within the FXS psychiatric literature over time to highlight the degree of female underrepresentation. By integrating findings across decades of research, this review aims to clarify the developmental course and prevalence of psychiatric symptoms in females with FXS and to strengthen the clinical understanding of their unique psychiatric vulnerabilities – ultimately informing more equitable and effective support.

## 2 Materials and methods

The systematic review and meta-analyses were pre-registered (37) prior to conducting the literature search and were carried out in accordance with the Preferred Reporting Items for Systematic Reviews and Meta-Analyses (PRISMA) guidelines (38).

### 2.1 Definition of psychiatric difficulties

This review examines the prevalence of both psychiatric conditions and psychiatric symptoms in females with FXS. A psychiatric condition – also referred to as a mental health condition – encompasses patterns of difficulties in cognitive, emotional, behavioral, and/or social functioning that adversely affect well-being and daily life (39). Such conditions are frequently underdiagnosed in the general population (40), with even higher rates of underdiagnosis reported among individuals with genetic syndromes, including FXS (7, 41). Symptoms associated with psychiatric conditions may be both experienced by the individual and observed by others familiar with them, even in the absence of a formal diagnosis (42). Assessing psychiatric symptoms, therefore, provides valuable insight into challenges experienced during the prodromal or subclinical stages of psychiatric conditions. Although Attention-Deficit/Hyperactivity Disorder (ADHD) is formally classified as a neurodevelopmental disorder, it is included in this review under psychiatric difficulties due to its clinical management within psychiatry and its frequent co-occurrence with other psychiatric conditions. For this reason, the term *psychiatric difficulties* – rather than *mental health difficulties* – is used throughout this review to synthesize findings across studies.

### 2.2 Search strategy

Literature searches were conducted in PsycINFO, MEDLINE, Embase, and Web of Science. The following inclusion filters were applied: (a) English language, (b) peer-reviewed publication, (c) publication date between 1980 and 2025, and (d) studies involving human participants.

Search strategies incorporated terms relating to psychiatric conditions recognized within standard diagnostic frameworks, including the Diagnostic and Statistical Manual of Mental Disorders (DSM; APA) and the International Classification of Diseases (ICD; WHO). Both subject headings (e.g., Medical Subject Headings, MeSH) and free-text keywords were adapted from a previous review examining psychiatric conditions and symptoms in genetic syndromes (43). A deliberately broad search approach was adopted, rather than applying *a priori* restrictions on which psychiatric difficulties might be relevant to the FXS phenotype, recognizing that much of the existing synthesis has been based primarily on male populations. That said, a key modification from the strategy used by Glasson et al. (43) was the inclusion of search terms related to ADHD, which were not part of their review. ADHD was explicitly incorporated in the present study to address the absence of prior systematic analyses of its presentation in females with FXS.

Terms related to autism were also included to explore potential associations between psychiatric difficulties and co-occurring autism in females with FXS. However, autism prevalence data were not synthesized independently, as this has been comprehensively reviewed elsewhere (29). Instead, autism prevalence was reported descriptively within the sample characteristics to provide contextual information. Similarly, ID-related terms were incorporated to investigate possible associations between intellectual ability and psychiatric outcomes. Due to limited available data, analyses of ID prevalence were beyond the scope of this review; thus, IQ information is also presented descriptively.

To ensure comprehensive coverage and allow assessment of sex representation across studies, no search terms were used to restrict results by sex or gender. The complete list of search terms is available in the **Supplementary Materials** (**Supplementary Table 1**).

### 2.3 Data extraction

**Figure 1:**
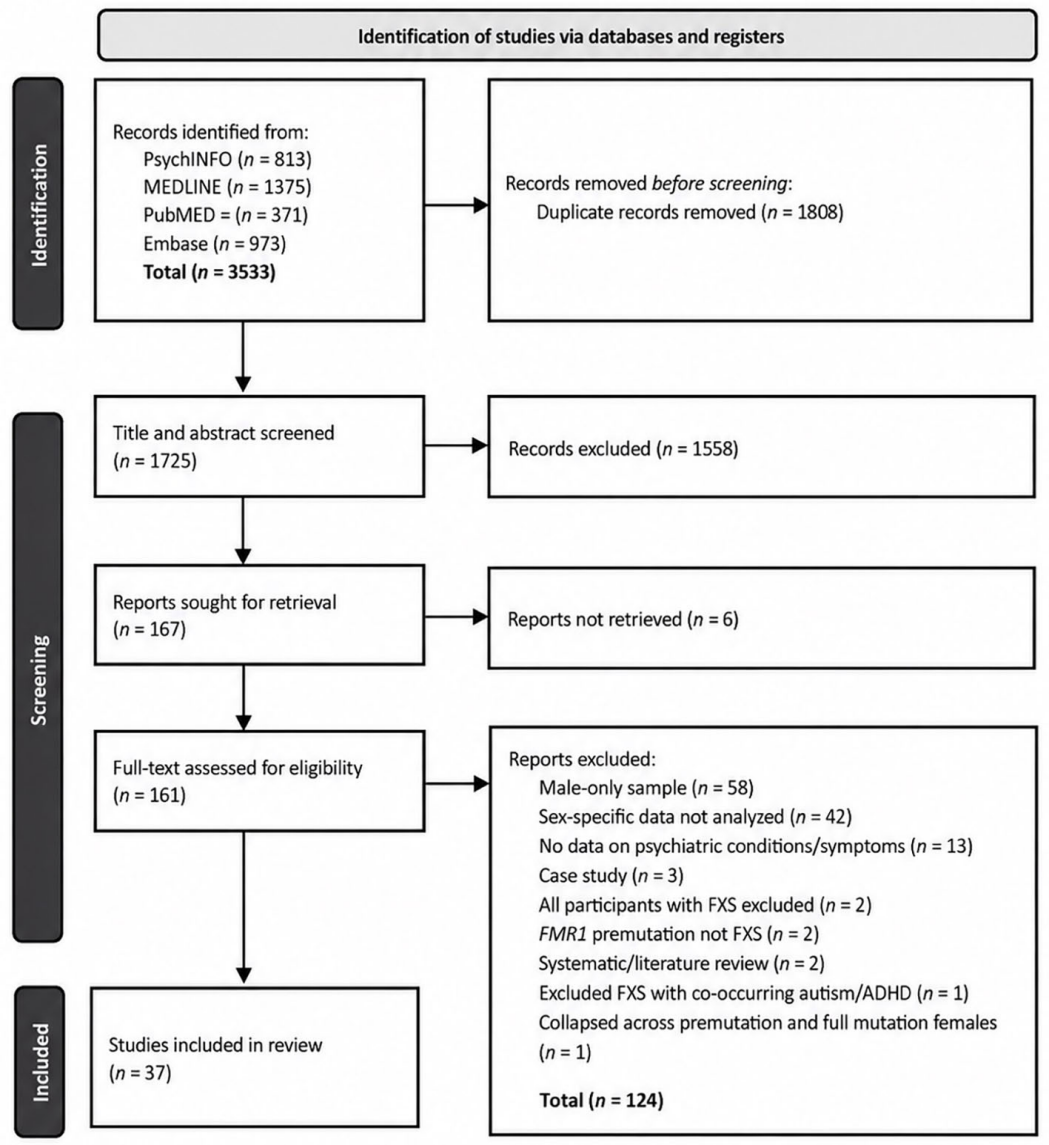
PRISMA 2020 flow diagram for systematic reviews.

The initial search yielded 3,533 studies (see **Figure 1**). After the removal of duplicates (*n =* 1,808), 1,725 articles remained and were independently screened by two authors (L.J. and C.K.) based on titles and abstracts, resulting in the exclusion of 1,558 studies. The remaining 167 articles underwent independent full-text review by both authors to determine eligibility. Inter-rater reliability demonstrated substantial agreement during the title and abstract screening (κ = 0.70) and almost perfect agreement during the full-text review (κ = 0.87). Any discrepancies between reviewers were discussed and resolved through consensus.

### 2.4 Inclusion and exclusion criteria

To be included in this review, studies were required to report either the prevalence of psychiatric diagnoses or the presence of psychiatric symptoms in females with FXS. The review encompasses research across childhood, adolescence, and adulthood. While it is ideal to examine these developmental stages separately, the rarity of FXS and the limited number of studies focused specifically on females necessitated the inclusion of research spanning the full lifespan.

Studies were excluded if participants carried the *FMR1* premutation rather than FXS (*n* = 2) or if findings were collapsed across these groups (*n* = 1), given that the premutation is associated with a distinct phenotype (6). Reports were also excluded if they focused exclusively on males (*n* = 58) or included females but did not analyze data by sex (*n* = 42) – a likely consequence of the limited resources available to support research that sufficiently captures variability among females.

### 2.5 Risk of bias and quality assessment

A risk-of-bias and quality assessment framework was adapted from tools used in previous meta-analyses on autism in genetic syndromes (44) and on females with FXS (29). While the criteria pertaining to sample identification and confirmation of genetic diagnosis were not changed, the assessment tool was modified to better reflect the evaluation of psychiatric difficulties specific to the aims of this review (see **Table 1**). Each study was rated on these three domains – sample identification, genetic syndrome confirmation, and assessment tool used – with scores ranging from 0 to 3, where higher scores indicate higher study quality and lower risk of bias. An average score was then calculated for each study to provide an overall quality index.

**Table 1:** Quality/risk of bias criteria for sample identification, confirmation of syndrome and assessment tool.

Criteria	Quality rating			
	Poor (0)	Adequate (1)	Good (2)	Excellent (3)
Sample identification	Sample source not specified or reported.	Single, restricted, or non-random sample (e.g., from a specialist clinic or previous research study)*.	Multiple restricted or non-random samples (e.g., sourced from multi-region specialist clinics).	Random or total population sampling.
Confirmation of syndrome	Syndrome confirmation not specified or reported.	Clinical diagnosis by a generalist (e.g., General Practitioner or Pediatrician).	Clinical diagnosis by an expert (e.g., Clinical Geneticist or Specialist Pediatrician).	Molecular, cytogenetic, or metabolic confirmation of diagnosis, including genetic report provided by the parent.
Assessment tool	Assessment tool not specified or reported.	Based on parent report and/or medical records; or an alternative assessment tool lacking established validity, such as a novel measure.	Clinical judgment using DSM or ICD diagnostic criteria without implementation of a formal assessment tool; or a validated broadband symptom scale (e.g., The Child Behavior Checklist [CBCL]; Anxiety, Depression, and Mood Scale [ADAMS]).	Clinical judgment using DSM/ICD diagnostic criteria informed by multimodal assessment; or a validated structured clinical interview aligned with DSM/ICD (e.g., Children’s Interview for Psychiatric Syndromes; P-ChIP); or a validated narrowband symptom scale (e.g., Conners Rating Scale).

*If individuals were recruited as part of a larger ongoing study, the recruitment strategy for that study was coded if described. If the strategy was not specified, it was automatically coded as 1, indicating that the sample originates from a single source (i.e., the larger ongoing study).

Two authors (C.K. and either R.H. or L.J.) independently evaluated all studies for quality and risk of bias. Inter-rater agreement was excellent across all domains, with weighted kappa values of 0.77 (95% CI: 0.51–1.00) for sample identification, 0.73 (95% CI: 0.49–1.00) for genetic syndrome confirmation, and 0.81 (95% CI: 0.60–1.00) for the assessment tool used. For overall quality scores, a two-way random-effects model using consistency and average-measures intraclass correlation (45) indicated a high level of agreement between raters (ICC = 0.85, 95% CI: 0.65–0.98). In instances of disagreement, the first author (L.J.) made the final determination of the ratings reported in this paper.

Consistent with Marlborough et al. (29) and prior methodological recommendations (46), no studies were excluded on the basis of quality or risk-of-bias scores, given the limited research available on this population. However, study quality was incorporated into the meta-regression analyses to assess whether it accounted for variability in prevalence estimates, as described below.

### 2.6 Data extraction and synthesis

Extracted data included study details (authors and country) and participant characteristics such as the sample size of females with FXS, age range (including mean and standard deviation where available), race/ethnicity, and socioeconomic status. When reported, information on IQ and co-occurring autism was also recorded. Details regarding exclusion criteria were collected to further characterize study samples and evaluate their representativeness.

Data on the prevalence of psychiatric difficulties were extracted along with information on the methods used to assess psychiatric symptoms and/or diagnoses. Prevalence estimates were derived from multiple sources, including diagnostic classifications based on established frameworks such as the International Classification of Diseases, 11th Revision (ICD-11; 47) and the Diagnostic and Statistical Manual of Mental Disorders, Fifth Edition (DSM-5; 48), caregiver-reported clinical diagnoses, medical records, and standardized symptom screening tools with validated clinical cut-offs (e.g., CBCL; 21). When the proportion of participants scoring above clinical thresholds on symptom screening measures was not provided (*n* = 12), study authors were contacted, resulting in additional data for two studies (49, 50). A narrative synthesis was then conducted to summarize key findings on psychiatric difficulties, encompassing symptom profiles, comparative prevalence rates, developmental trajectories, and associations with intellectual ability and autism.

### 2.7 Meta-analyses

Meta-analyses were conducted in R using the metafor package (v6.9) (51) to estimate the prevalence of clinically significant psychiatric difficulties in females with FXS, where sufficient data were available. For each study, the total number of females with FXS and the number of cases (i.e., females diagnosed with a psychiatric condition) were extracted to calculate proportions. Because some studies reported data across multiple psychiatric domains, an overall pooled prevalence and between-domain comparisons were not conducted. Instead, separate meta-analyses were performed for each psychiatric domain.

The primary analytic approach employed a random-effects inverse-variance meta-analysis of logit-transformed proportions, yielding pooled logit estimates with corresponding confidence intervals. Results are presented alongside study-level data, including sample size, number of cases, assessment tools, and quality ratings (see **Table 2**). Pooled logit estimates were back-transformed to percentages with 95% confidence intervals (CIs) for ease of interpretation. Between-study variance (τ²) was estimated using the DerSimonian–Laird method, and heterogeneity was quantified using I², with values below 75% interpreted as indicating negligible to moderate heterogeneity (97).

**Table 2:** Overview of reviewed studies.

Author (year) and country of study	Sample characteristics				Psychiatric outcome		Quality rating			
	Sample size (n)	Age in years (M ± SD) (range)	IQ (M ± SD)	% autism (assessment tool)	Psychiatric condition(s) and/or symptoms measured	Psychiatric assessment tool	Sample	Genetic	Assessment	Average score
Aman et al. (52) USA	154	19.13 ± 11.04 (2 - 24)	NR	NR	Irritability, social withdrawal, hyperactivity	ABC	2	3	2	2.33
Arpone et al. (53) Australia	21	7.50 ± 3.12 (3 -17)	64.10 ± 13.92	NR	Self-injury, hyperactivity, tantrums/aggressive behaviors	ABC	2	3	2	2.33
Bailey et al. (54) USA	259	0–11: 45%, 12 - 18: 24%, 19 - 30: 20%, >30: 11%	NR	6%	Attention problems, hyperactivity, aggressiveness, self-injurious behavior, anxiety, depression	Parent reported diagnosis	2	0	1	1.00
Bailey et al. (55) USA	299	16.30 (SD & range NR)	NR	NR	Attention problems, anxiety, hyperactivity, mood swings, anger or aggression, depression, self-injury	Medication use reported by parents to manage symptoms	2	0	1	1.00
Baker et al. (2019) Australia & Chile	28	10.90 ± 9.23 (2 -34)	64.5 ± 17.3	71% (ADOS-2)	Irritability, lethargy, stereotypy, hyperactivity, social avoidance	ABC-C	2	3	2	2.33
Bartholomay et al. (56) USA	65	10.59 ± 3.00 (6 -16)	NR	46% (ADOS-2)	Social anxiety	ADAMS; CBCL	2	3	2	2.33
Chromik et al. (57) USA	33	20.40 ± 2.94 (15 -25)	NR	NR	ADHD	ADHD-T; ABC-C	1	3	3	2.33
Cordeiro et al. (9) USA	39	12.35 ± 6.17 (5 -33)	77.2 ± 20.65	28.2% (ADOS-2, ADI-R, DSM-IV)	Separation anxiety, social phobia, specific phobia, panic disorder, agoraphobia, general anxiety disorder, obsessive compulsive disorder, post-traumatic stress disorder, selective mutism	ADIS-IV; ADAMS	1	3	3	2.33
del Hoyo Soriano et al. (58) USA	16	12.00 ± 1.50 (10 -15)	NR	NR	Anxious/Depressed & Withdrawn/Depressed	CBCL	2	3	2	2.33
Freund et al. (59) USA	17	12.80 ± 7.50 (4 -27)	78.2 ± 23.8	NR	ADHD, separation anxiety disorder, avoidant personality disorder, overanxious disorder, mood disorder (general), major depression, dysthymia, depression – not otherwise specified	DICA-P	2	3	3	2.67
Gao et al. (60) USA	56	10.62 ± 2.93 9 (6 - 16)	81.43 ± 17.60 (verbal IQ)	NR	Depressed mood, general anxiety, social anxiety	ADAMS; CBCL	2	3	2	2.33
Hagerman et al. (13) USA	32	8.00 ± 4.62 (1 -18)	80.4 ± 18.6	NR	ADHD	Conners Rating Scale (Conners, 1973)	1	3	3	2.33
Hall et al. (11) USA	29	13.06 ± 3.93 (5 - 20)	70.76 ± 20.91	45% (ADOS-G)	Compulsive behavior, self-injurious behavior	CBC, SIB-C	2	3	3	2.67
Hartley et al. (28) USA	89	30.27 ± 7.76 (22 - 64)	NR	9.64% (PR)	Inattention, hyperactivity, aggression, self-injury, affect problems (anxiety/depression)	Parent reported diagnosis	2	0	1	1.00
Hessl et al. (61) USA	40	10.42 ± 3.10 (range NR)	75.48 ± 22.30	NR	Withdrawal, somatic complaints, anxiety/depression, thought problems, attention problems, delinquent behavior, aggressive behavior, internalizing problems, externalizing problems	CBCL	2	3	2	2.33
Jordan et al. (62) USA	47	11.84 ± 3.11 (6 - 18)	78.27 ± 17.96 (verbal IQ)	48% (ADOS-2)	Depression, general anxiety, social anxiety, withdrawn/depressed, somatic complaints	ADAMS; CBCL	2	3	2	2.33
Lachiewicz et al. (63) USA	60	7.21 (3 - 13)	Median = 77.5, Range = <50 - 114	NR	ADHD, conduct problems, psychosomatic, impulsive-hyperactive, anxiety and hyperactivity	Conners’ Parent’s Questionnaire (Conners, 1990)	2	3	3	2.67
Lesniak-Karpiak et al. (64) USA	21	15.53 ± 4.22 (7 - 22)	97.4 ± 15.96 (<11 years), 92.00 ± 16.36 (>11 years)	NR	Anxiety, social anxiety, withdrawal	CBCL; RCMAS; SPAI-C	2	3	3	2.67
Li et al. (65) USA	35	11.35 ± 3.07 (range NR)	82.40 ± 16.79 (verbal IQ)	NR	General anxiety, social anxiety	ADAMS	2	3	2	2.33
Li et al. (66) USA	32	11.76 ± 2.93 (range NR)	81.97 ± 17.75 (verbal IQ)	NR	Depressed mood, general anxiety, social anxiety, anxious/depressed	ADAMS; CBCL	2	3	2	2.33
Lightbody et al. (67) USA	58	10.58 ± 2.98 (6 - 16)	81.93 ± 17.50 (verbal IQ)	NR	General anxiety, depressed mood, social anxiety, withdrawn/depressed	ADAMS; CBCL; PARS-R	2	3	3	2.67
Lonzano et al. (68) USA	87	2 - 5: 5%, 6 - 12: 20%, 13-17: 22%, 18 - 29: 34%, 30 -49: 18%, 50+: 1%.	NR	NR	Anxiety, avoidance, aggression, self-injury, hyperactivity	Novel survey	2	0	1	1.00
Mazzocco et al. (69) USA	30	10.58 ± 3.17 (6 – 16)	87.4 ± 14.1	3.33% (NDI)	Anxiety	NDI; Anxiety Composite score (as described by Freund et al., 1997)	2	3	3	2.67
Miller et al. (2021) USA	43	10.58 ± 2.86 (6 - 16)	79.70 ± 17.93 (verbal IQ)	42% (ADOS-2)	General anxiety, separation anxiety/phobias, social anxiety, obsessions/compulsions	MASC	2	3	3	2.67
Muller et al. (70) USA	11	9.5 (8 - 10)	NR	19% (PR)	Defiance, hyperactivity/inattention, tantrum, aggression, anxiety, self-injury	Qualitative interview with diagnosis and/or symptoms described by parent coded	2	0	1	1.00
Reilly et al. (41) UK	21	11.58 ± 3.60 (4 -19)	NR	14% (PR)	ADHD or a mental health condition	Parent reported diagnosis	2	0	1	1.00
Reisinger et al. (71) USA	42	16.51 ± 10.55 (2 - 50)	66.0 ± 19.51 (verbal IQ)	NR	Irritability, withdrawal, hyperactivity	ABC	2	3	2	2.33
Roberts et al. (49) USA	15	3.77 ± 0.90 (6–72 months)	57.76 ± 20.13 (non-verbal DQ)	29% (ADOS-2; PAPA)	Separation anxiety, social phobia, specific phobia, generalized anxiety disorder	PAPA	2	3	3	2.67
Russo-Ponsaran et al. (72) USA	16	16.06 ± 7.19 (5 - 35)	61.33 ± 12.74 (*n =* 6)	NR	Anxiety	PARS-R; ADAMS; ABC-C; CGI-S	2	3	3	2.33
Shaffer et al. (73) USA	11	19.96 ± 8.47 (5 - 36)	NR	NR	Irritability, withdrawal, hyperactivity manic/hyperactive, depressed mood, social anxiety, generalized anxiety	ADAMS	1	3	2	2.00
Sullivan et al. (74) USA	6	9.30 ± 1.90 (7 -13)	83.7 ± 15.6	20% (CARS)	ADHD	CSI-PC; ASI-PC; CSI-TC; ASI-TC; CBCL	2	0	2	1.33
Symons et al. (12) USA	51	15.90 ± 10.90 (range NR) (reported for full sample; 90% male)	NR	34% (PR)	Attention problems, hyperactivity, aggression, anxiety, depression, self-injurious behavior	Parent Report (survey)	2	0	1	1.00
Visootsak et al. (75) USA	18	6.6 (SD NR) (1 - 17)	NR	12% (MR)	ADHD, anxiety, aggression	Medical records from specialist FXS clinic	1	3	1	1.67
Wall et al. (50) USA	11	4.23 ± 0.53 (6–60 months)	86.73 ± 18.46 (DQ)	NR	General anxiety	SCAS-P	2	3	3	2.67
Wheeler et al. (76) USA	58	17.10 (SD NR) (6 -44)	NR	NR	Anxiety, ADHD, irritability, hyperactivity, social avoidance,	ABC-C; ADAMS; CASI/AI	1	1	3	1.67
Wheeler et al. (77) USA	132	16.33 ± 9.85 (3 -47)	NR	25% (DSM-IV)	Hyperactivity, anxiety, aggression, sensory issues	Novel survey	2	0	1	1.00

NR = not reported (includes when reported for whole sample but not females specifically). MR = diagnosis from medical records. PR = parent reported diagnosis. ABC = Aberrant Behavior Checklist (20); ABC-C = Aberrant Behavior Checklist – Community Version (78); ADAMS = Anxiety, Depression, and Mood Scale (19); ADHD-T = ADHD Test (79); ADIS-IV = Anxiety and Related Disorders Interview Schedule for DSM-5 (80); ADOS-2 = Autism Diagnostic Observational Schedule – Second Edition (81); ADOS-G = Autism Diagnostic Observational Schedule – Generic (82); AI = Adult Inventories (83); ASI-PC = Adolescent Symptom Inventory-4: Parent Checklist (84); ASI-TC = Adolescent Symptom Inventory-4: Teacher Checklist (84); CARS = The Childhood Autism Rating Scale (85); CASI = Child and Adolescent Symptom Inventory (86); CBCL = Child Behavior Checklist (21); CGI-S = Clinical Global Impression-Severity Scale (87); CBC = Compulsive Behavior Checklist (88); CSI-TC = Childhood Symptom Inventory-4: Teacher Checklist (84); CSI-PC = Childhood Symptom Inventory-4: Parent Checklist (84); DICA-P = Diagnostic Interview for Children and Adolescents-Parent version (89); MASC = Multidimensional Anxiety Scale for Children (90); NDI = Neuropsychiatric Diagnostic Interview (90); PAPA = Preschool Age Psychiatric Assessment (91); PARS-R = Pediatric Anxiety Rating Scale– Revised (92); RMCAS = Revised Children’s Manifest Anxiety Scale (93); SIB-C = Self-Injury Checklist (94); SCAS-P = Spence Children’s Anxiety Scale for Parents (95); SPAI-C = Social Phobia and Anxiety Inventory for Children (96).

To assess potential sources of variability in prevalence estimates, meta-regression analyses were conducted, examining whether study quality contributed to heterogeneity. To account for differences in participant age ranges, studies were coded based on the breadth of their age distribution: those restricted to a single developmental stage (e.g., preschool, school-age, or adult-only) were classified as narrow, while those spanning multiple developmental stages (e.g., preschool through adulthood) were classified as wide. This categorical approach was adopted because several studies reported age in ranges or categorical form, precluding the use of age as a continuous moderator. The age-based moderator was then used in subgroup analyses to evaluate whether more homogeneous versus heterogeneous age samples influenced prevalence estimates.

## 3 Results

This section begins with an overview of sex representation in research on psychiatric difficulties in FXS to date, followed by a summary of the final pool of included studies (*n =* 37) that described females. **Table 2** presents the sample characteristics, psychiatric outcomes, and quality ratings for these studies. The section then provides a narrative synthesis and meta-analytic findings for the most commonly reported psychiatric difficulties - anxiety, depression, ADHD, aggression, and self-injury – and concludes with a description of whether these difficulties relate to intellectual ability and autism.

### 3.1 Sex representation in research on psychiatric difficulties in FXS

**Figure 2:**
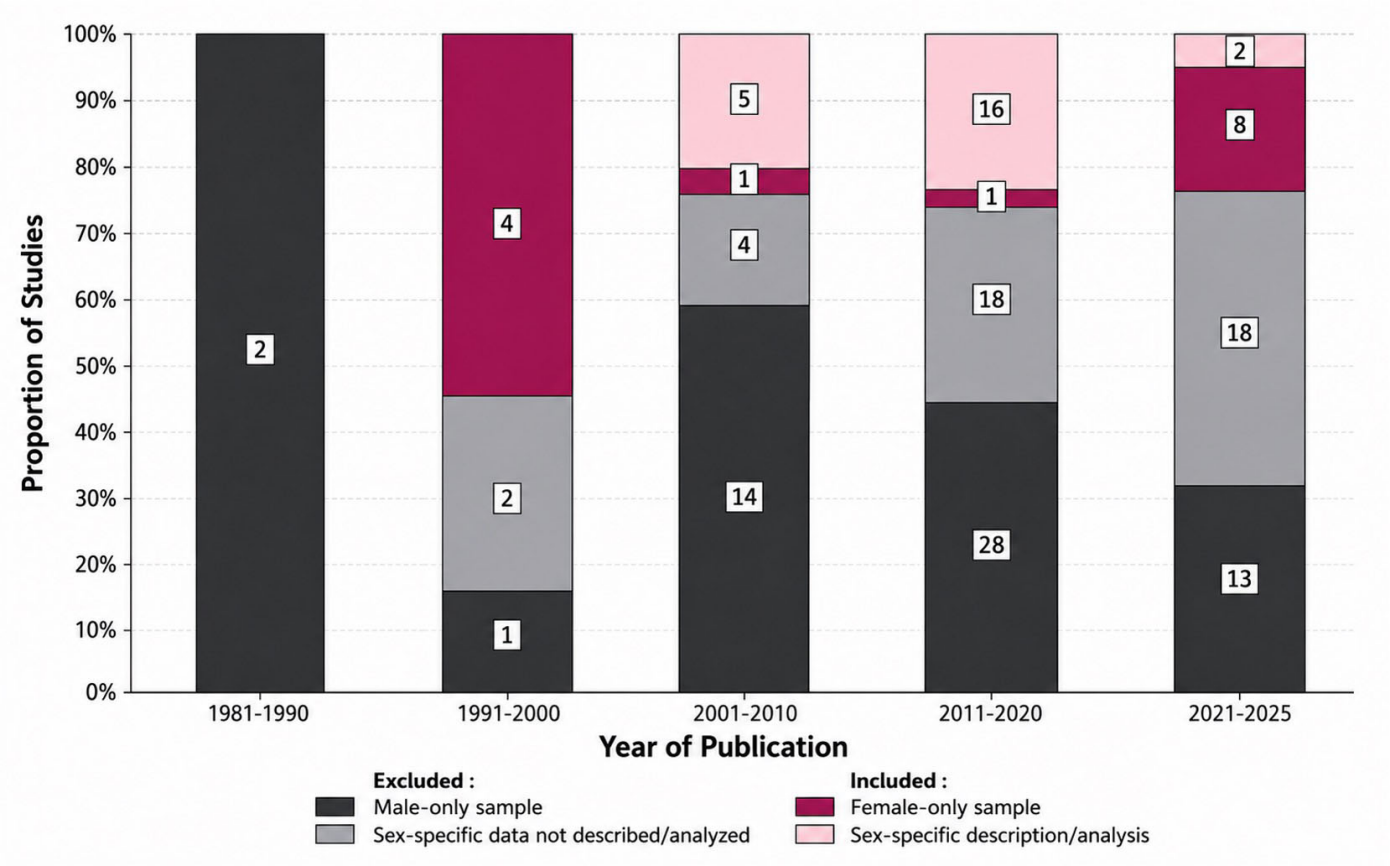
Trends in sex representation among studies on psychiatric difficulties in fragile X syndrome.

Among the studies included in this review, 23 conducted sex-specific analyses (i.e., analyzed males and females separately) within mixed-sex samples, while 14 focused exclusively on females. In contrast, 58 studies were excluded because they included only male participants. Overall, this pattern indicates that research on males has historically outnumbered that on females by approximately 4:1 – meaning that for every study investigating psychiatric difficulties in females with FXS, about four have focused on males. As shown in **Figure 2**, however, recent years reveal a positive shift toward greater balance, now approaching a ratio of 2:1, underscoring the relevance and timeliness of the present review.

### 3.2 Characteristics of the reviewed studies

Across the 37 studies reviewed, participants’ mean ages ranged from 3.77 years (49) to 30.27 years (28). The majority of studies (89%; *n =* 33) were conducted in the United States, with two based in Australia (53, 98), one in Chile (Baker et al., 2019), and one in the United Kingdom (41). Reporting on participants’ race and ethnicity was generally limited; of the studies that included such data (24%; *n =* 9), most samples were primarily White/Caucasian and non-Hispanic (52, 53; Baker et al., 2019; 9, 28, 56, 58, 62, 74). Similarly, socioeconomic indicators such as annual household income were rarely reported – among the few studies that did include such data (11%; *n =* 4), substantial variability prevented meaningful interpretation (52, 54–56, 58, 59, 61). Regarding exclusion criteria, four studies (11%) explicitly excluded participants with psychosis or bipolar disorder (56, 62, 67, 99), while one excluded individuals with significant language or cognitive impairments (58).

#### 3.2.1 Intellectual ability and autism co-occurrence in reviewed samples

Among the 13 studies that provided full-scale or verbal IQ scores, mean values ranged from 61.33 (72) to 97.40 (64). Autism prevalence was also reported in 12 studies, with rates spanning from 10% (28) to 48% (62). An earlier study (69) reported a substantially lower prevalence of 3%, which reflected the narrower DSM-III diagnostic criteria for “autistic disorder” (100).

#### 3.2.2 Psychiatric conditions and symptoms represented

The reviewed literature provided sufficient data to describe several key areas of concern. Among these, anxiety was the most frequently described, appearing in 22 studies (59%) that described anxiety disorders or related symptoms (9, 55, 56, 58–67, 69; Miller et al., 2021; 12, 49, 50, 73, 75, 76, 101). Depression and mood-related difficulties were also reported in 13 studies (33%), ranging from formal diagnoses to subclinical symptoms such as dysthymia, mood swings, and withdrawn or depressed affect (9, 55, 58–62, 66, 67; Miller et al., 2021; 12, 73, 101). ADHD and related symptoms – such as hyperactivity and inattention – were described in 13 studies (33%; 12, 13, 41, 53, 55, 57, 59, 61, 63, 70, 74–76). Aggression, including conduct-related problems, was described in 8 studies (22%) (12, 53, 55, 61, 63, 70, 75, 77), and self-injurious behaviors were noted in 6 studies (16%; 11, 12, 53, 55, 70, 75).

### 3.3 Meta-analytic prevalence estimates and narrative review within psychiatric domains

The following sections present meta-analytic prevalence estimates based on studies with sufficient data (see **Supplementary Tables 2, 3**), complemented by a narrative review of symptom profiles across all included studies. Notably, the majority of these studies (72.2%) achieved average quality ratings above 2.3, underscoring the overall methodological rigor of the reviewed literature.

#### 3.3.1 Anxiety

**Figure 3:**
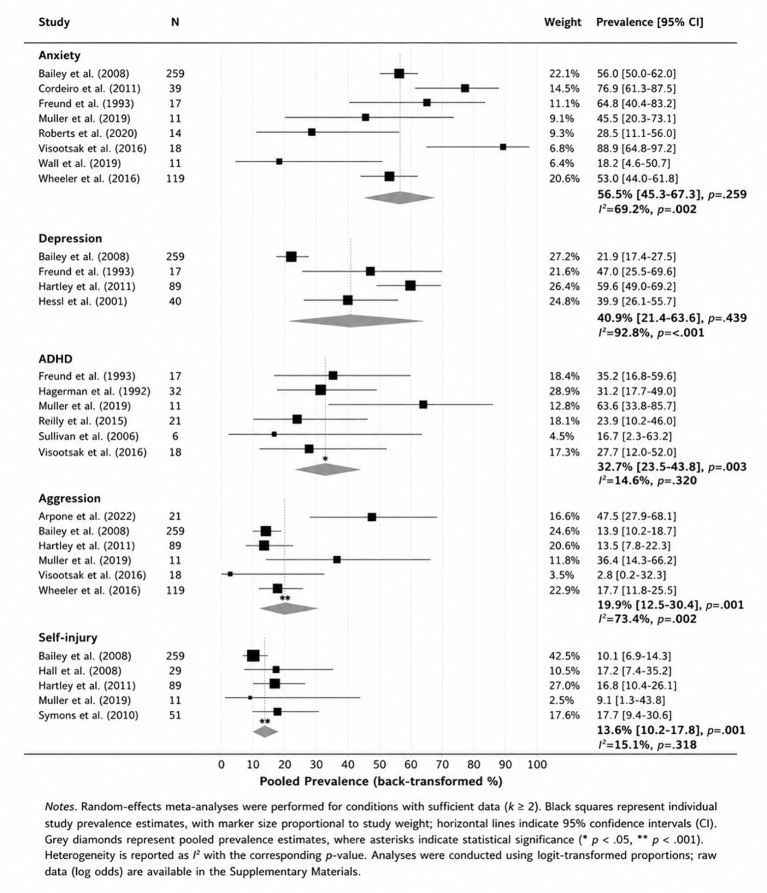
Pooled prevalence estimates for psychiatric difficulties in females with fragile X syndrome. Random-effects meta-analyses were performed for conditions with sufficient data (k ≥ 2). Black squares represent individualonfidence intervals (CI). study prevalence estimates, with marker size proportional to study weight; horizontal lines indicate 95% confidence inteGrey diamonds represent pooled prevalence estimates, where asterisks indicate statistical significance (* p < .05, ** p < .001), Heterogeneity is reported as /2 with the corresponding p-value. Analyses were conducted using logit-transformed proportions; raw data (log odds) are available in the Supplementary Materials.

**Meta-analysis** of eight studies (*k =* 8, *n =* 488) estimated the pooled prevalence of at least one anxiety disorder in females with FXS at 56.5% (95% CI: 45.2–67.3%). Although this estimate did not reach statistical significance (*p = .*259), moderate heterogeneity (I² = 69.2%, τ² = .228, *p = .*002) suggests that the lack of significance likely reflects considerable variability across studies, which reported rates ranging from 18.2% (50) to 88.9% (75). Study quality did not significantly influence prevalence estimates (*p = .*422). Subgroup analyses revealed age-related differences (Q = 9.32, *p <*.001): studies including both children and adults (*k =* 5) reported a higher pooled prevalence (63.8%, 95% CI: 52.8–73.5%) with moderate heterogeneity (I² = 68.9%, τ² = .151, *p = .*012), whereas studies restricted to preschool- or school-aged samples (*k =* 3) found lower prevalence (31.6%, 95% CI: 18.2–48.9%) and minimal heterogeneity (I² = 0%, τ² = 0, *p = .*390). Despite the variability, anxiety emerged as the most prevalent psychiatric difficulty across all domains assessed (see **Figure 3**).

**Narrative synthesis** of the 22 reviewed studies further supports that anxiety disorders and related symptoms are among the most widely examined psychiatric difficulties in females with FXS. One of the earliest investigations, Freund et al. (59), identified increased vulnerability to anxiety – particularly social anxiety-relative to IQ-matched peers. Subsequent studies have consistently reported elevated anxiety symptoms in females with FXS, both compared with unaffected females and, in some instances, males with FXS. Reported anxiety presentations include generalized anxiety (9, 50, 54, 60, 65, 66, 76), social anxiety (9, 49, 59, 60, 64–66, 69, 71, 72), separation anxiety (9, 49), selective mutism (9), and specific phobias (9, 61; Miller et al., 2021; 49).

Females with FXS present with anxiety through withdrawal, refusal, crying, and internalizing behaviors (68, 72, 73, 76). Symptoms of generalized and social anxiety specifically have been observed as early as the preschool years (49, 50). However, at this age, females are less likely than age-matched males to meet diagnostic criteria for multiple DSM-5 anxiety disorders (0% vs. 13%; 49). By adulthood, however, multiple anxiety diagnoses are common in both sexes, affecting approximately 55% of females and 60% of males (9). In their comprehensive DSM-IV assessment, Cordeiro et al. (9) found that the most prevalent anxiety disorders among females were social phobia (55%) and specific phobia (51%), followed by selective mutism (21%), separation anxiety (18%; roughly three times more common than in males), and generalized anxiety (18%). Obsessive–compulsive disorder (OCD) was also identified in 18% of females, though it should be noted, OCD is now categorized separately from anxiety disorders under DSM-5.

Rates of medication use for anxiety among females with FXS appear to rise steadily across cohorts – from 9% in early childhood (0–5 years), 28% in middle childhood (6–10 years), 31% in adolescence (11–15 years), and up to 40% in adulthood – rates comparable to those observed in males (55). However, it remains unclear which anxiety presentations are most likely to receive treatment. Similarly, in community settings, the presentations most likely to be diagnosed remain uncertain. Specialty clinic evaluations have identified anxiety in 87% of females compared with 62% of males, whereas community-based diagnoses are markedly lower (11% vs. 5%), suggesting substantial under-diagnosis more generally (75).

Longitudinal research suggests that social anxiety is the key concern to monitor as females with FXS age. While generalized anxiety symptoms remain relatively stable-though elevated-throughout childhood and adolescence, social anxiety tends to rise sharply during puberty. This developmental trajectory distinguishes females with FXS from other neurodivergent females of a similar age (56, 67). Some evidence indicates that social anxiety may plateau in adulthood for females with FXS relative to males (52). However, because males present with ID and have lower levels of social independence (28), this apparent difference may reflect differences in social opportunities rather than inherent sex-based variation in anxiety expression. Thus, any apparent plateau in adulthood should be interpreted cautiously, especially given the significant impact of affect problems on adult independence in females with FXS (28).

#### 3.3.2 Depression

**Meta-analysis** of four studies (*k =* 4, *n =* 405) estimated the pooled prevalence of depression in females with FXS at 40.9% (95% CI: 21.5–63.8%). However, this estimate was not statistically significant (*p = .*439) and exhibited very high heterogeneity (I² = 92.8%, τ² = .808, *p <*.001), reflecting considerable variability among studies (see **Figure 3**). Meta-regression analyses revealed no significant effect of study quality (*p = .*519), and subgroup analyses by age were not feasible since all studies included mixed samples of children and adults.

**Narrative synthesis** identified depression and mood-related symptoms across 13 studies. Some evidence suggested that females with FXS exhibit higher levels of depressive symptoms than age- and IQ-matched female peers (59, 66), while other research found no significant differences (69), particularly when compared against other neurodivergent females (56, 60, 67). However, what appears to distinguish the depressive profile in females with FXS is its co-occurrence with social withdrawal symptoms, with both domains tending to peak around puberty – a pattern that was not observed in the neurodivergent female comparison group (67).

Although direct comparisons with males remain limited, existing data indicate that females with FXS display higher parent-reported depressive symptoms than males (12), particularly regarding internalizing difficulties, such as feelings of worthlessness, loneliness, and frequent crying (61). A large parent survey also found that adult females were nearly twice as likely as males to have been diagnosed with or treated for depression (22% vs. 12%; 54). However, a later study found similar rates of medication use for depression across sexes (females: 8%; males: 9%; 55), suggesting that while females may be more vulnerable to depression, they are not more likely to receive treatment. This is concerning given that affect problems in females have been linked to reduced adult independence, an association not observed in males (28).

#### 3.3.3 Attention-deficit/hyperactivity disorder

Meta-analysis of six studies (*k =* 6, *n =* 105) estimated the prevalence of ADHD in females with FXS at 32.8% (95% CI: 23.5–43.8%), which was statistically significant (*p = .*003) and showed low heterogeneity (I² = 14.6%, τ² = .050, *p = .*320), suggesting a stable and reliable estimate (see **Figure 3**). Meta-regression analyses showed that study quality had no significant impact on prevalence estimates (*p = .*391), and subgroup analyses by age revealed no significant differences between studies including both children and adults (*k =* 4) and those focusing solely on childhood samples (*k =* 2) (*p = .*63).

Narrative review of 13 studies identified ADHD and related symptoms are the second most extensively investigated psychiatric difficulty in females with FXS. Parent-reported data indicate that ADHD is more prevalent in females with FXS (24%) than in females with other genetic syndromes, including Prader–Willi (2%), Williams (3%), and velo-cardio-facial syndrome (6%). Within FXS, parent-reported ADHD rates are similar between females (24%) and males (22%; 41), although clinical evaluations in FXS specialty settings indicate higher rates in males (51%) compared to females (29%) (75). Collectively, these findings indicate that ADHD is a defining feature of the psychiatric profile in females with FXS – more prevalent than in other genetic syndromes but typically less pronounced than in males.

Research exploring the developmental trajectory of ADHD symptoms in females with FXS remains limited. One study found that no preschool-aged females with FXS met diagnostic criteria, compared with 31% of age-matched males, suggesting that symptoms may be less apparent or impairing in early childhood (49). Although both sexes exhibit a peak in hyperactivity around ages 5–6, this peak tends to be shorter-lived in females, whose symptoms decline more rapidly than those of males, who often continue to display persistent hyperactivity into middle childhood (52). This more transient symptom pattern in females may contribute to under-identification, as teachers are less likely than parents to report inattention or hyperactivity – a discrepancy not observed in males – highlighting the potential for ADHD symptoms in females to be overlooked during childhood (74).

Early studies suggested that ADHD might be underdiagnosed in females with FXS, proposing that attention deficit disorder without hyperactivity (now ADHD–Inattentive Type in DSM-5) may better capture the presentation in some cases (13, 59). Later research supports this notion: females with FXS typically show more pronounced inattentive symptoms compared with both mental-age–matched peers (74) and unaffected biological siblings (61). While inattention rates are broadly similar between sexes, hyperactivity is less pronounced in females (12, 54, 71, 73, 76), especially among those with mosaicism, whereas males display elevated hyperactivity regardless of mosaic status (Baker et al., 2019). This sex difference is also reflected in treatment patterns: females are more likely to receive medication for inattention (23%) than for hyperactivity (11%), while males are treated for both symptom domains at similar rates (55). By adulthood, inattention emerges as the predominant challenge for females with FXS (66% vs. 28% for hyperactivity), though both remain less prevalent than in males (inattention: 83%; hyperactivity: 64%) (28).

#### 3.3.4 Aggression

Meta-analysis of six studies (*k =* 6, *n =* 517) estimated the prevalence of aggression in females with FXS at 20.0% (95% CI: 12.5–30.4%; *p <.001*), a statistically significant finding, but with substantial heterogeneity (I² = 73.4%, τ² = .299, *p = .*002; see **Figure 3**). Meta-regression analyses revealed that study quality significantly predicted this variability (β = –2.83, *p = .*01; OR ≈ 0.06), with higher-quality studies reporting notably lower prevalence estimates. Subgroup analyses by age were not possible, as all included studies involved mixed samples of children and adults.

Narrative synthesis indicated that although aggression is not the most prevalent psychiatric feature in females with FXS, it is frequently reported (*n =* 8), often in studies primarily focused on males with females included as comparison groups. Across studies, aggression consistently appears less common in females than in males (12, 70, 77), and this sex difference persists into adulthood (28). When aggression does occur in females, it typically manifests as defiance (91%) and temper tantrums (73%), rather than verbal (46%) or physical aggression (36%; 70). In older female cohorts, defiance (70%), temper tantrums (65%), and arguing (60%) remain common, suggesting that oppositional behaviors persist into adulthood (77). Around 13% of females were reported to have injured a caregiver (e.g., hitting, knocking down, slapping) and 14% a peer within the past year, though these estimates are based on caregiver reports and may not align with clinically diagnosed aggression-related disorders such as conduct disorder or oppositional defiant disorder.

Clinically, conduct disorder was reported in 18% of females with FXS in an early study (59), whereas more recent findings from a specialty FXS clinic identified no females with clinically significant aggression compared to 25% of males (75). These results underscore sex-related differences in aggression expression, with oppositional and defiant behaviors (e.g., refusal, tantrums) being more characteristic of females, in contrast to the physical aggression more often seen in males and more likely to prompt clinical intervention. Importantly, emerging evidence suggests that oppositional behaviors in females with FXS may reflect underlying anxiety (68), consistent with findings linking anxiety severity to aggression in females with FXS (77). This highlights the potential for targeting anxiety symptoms as a pathway to reducing aggression-related behaviors in females with FXS.

#### 3.3.5 Self-injury

**Meta-analysis** of five studies (*k =* 5, *n =* 439) found that self-injury is the least prevalent psychiatric difficulty among females with FXS, with a pooled prevalence of 13.5% (95% CI: 10.2–17.8%), that was statistically significant (*p <*.001) with low heterogeneity (I² = 15.1%, τ² = .022, *p = .*318; see **Figure 3**). Meta-regression analyses identified a significant effect of study quality (β = –2.23, *p = .*01; OR ≈ 0.11), indicating that higher-quality studies predicted considerably lower prevalence rates. Subgroup analyses by age could not be conducted because all included studies comprised mixed samples of children and adults.

Narrative synthesis of six studies similarly found that self-injury in females with FXS is reported at relatively low rates. Both parent and clinician reports indicate it is one of the least frequently endorsed psychiatric concerns (53, 70). During childhood and adolescence, approximately 17% of females engage in self-injury compared with 58% of males (11). By adulthood, prevalence estimates decrease to 10–17% in females and 41–47% in males (28, 54). Medication use follows a similar pattern, with 11% of males and 4% of females receiving medication for self-injury at some point in their lifetime (55).

In both sexes, the likelihood of self-injury increases with the presence of developmental delays and multiple co-occurring conditions (54) – notably, co-occurring anxiety in females and hyperactive or impulsive ADHD symptoms in males (12). These associations may underlie sex-specific patterns of self-injurious behavior: hitting (16%) and biting (42%) are more common in males than in females (0% and 7%, respectively). In contrast, rubbing or scratching behaviors are the most frequently observed forms of self-injury in females (14%; 12).

### 3.4 Associations between intellectual ability, autism, and psychiatric difficulties

Available evidence indicates that intellectual ability is not significantly associated with anxiety (59, 64, 69), anxiety-related medication use (55), compulsive behaviors (11), or ADHD symptoms (64) in females with FXS. In contrast, co-occurring autism and higher levels of autistic traits have been consistently linked to greater psychiatric difficulties. Specifically, elevated autistic traits have been associated with increased generalized anxiety, social anxiety, and depressive symptoms (56, 67, 69), and females with both FXS and autism are significantly more likely to use medications targeting anxiety, depression, and aggression (55). Additionally, self-injurious behaviors occur far more frequently among females with FXS and co-occurring autism (40%) than among females with FXS – only (9%) (12, 70). Findings regarding ADHD symptoms are more mixed. These symptoms have been linked to challenges with adaptive social functioning across age but not specifically to autistic traits (57), and rates of medication use for inattentive or hyperactive behaviors do not differ based on the presence of co-occurring autism (55).

## 4 Discussion

The presentation of FXS in females has long been underexamined, with their clinical needs often mischaracterized as “milder”. To challenge this misconception, the present systematic review and meta-analyses investigated psychiatric difficulties in females with FXS, exploring their associations with intellectual ability and autism. It also provides the first systematic description of sex representation in research on psychiatric outcomes in FXS. Although studies involving males have outnumbered those on females by approximately 4:1, the existing body of research on females with FXS offers substantial and consistent evidence of marked psychiatric vulnerability. Across 37 studies and accompanying meta-analyses, elevated rates of psychiatric difficulties were observed, with prevalence estimates exceeding those seen among females in the general population. Anxiety was most prevalent (57%), followed by depression (41%) and ADHD (33%). Although less common, aggression (20%) and self-injury (14%) still affected a notable subgroup. Crucially, co-occurring autism was consistently associated with heightened anxiety, depression, aggression, and self-injury, while intellectual ability showed no clear relationship with psychiatric difficulties. These findings highlight the urgent need for improved recognition, early identification, and targeted mental health support for females with FXS. The ongoing underrepresentation of females with FXS in research suggests that current estimates may underestimate the true scope of their psychiatric difficulties. Progress will require moving beyond the assumption that intellectual disability represents the primary clinical concern in FXS and incorporating insights from research on neurodivergent females more broadly.

### 4.1 Emergence of anxiety and depression

Current evidence suggests that females with FXS exhibit substantially higher rates of anxiety and depression than females in the general population. Meta-analytic estimates indicate that roughly 57% of females with FXS meet criteria for at least one anxiety disorder — far exceeding the 23% prevalence reported for females in the National Comorbidity Survey (102). Depression follows a similar pattern: the current meta-analysis estimates a prevalence of 41% among females with FXS, compared with 25% among females in the general population (102). Although these epidemiological benchmarks are dated, they represent the most recent U.S. diagnostic estimates available and align with the studies included in the present meta-analysis, all of which collected prevalence data prior to the COVID-19 pandemic (1993–2020). This context is important: even before the pandemic, rates of anxiety and depression in females with FXS were nearly double those observed in the general population. Since then, anxiety and depressive symptoms have risen sharply and remain elevated worldwide, with female sex consistently identified as a risk factor for poorer mental health outcomes in the general population (103). Given evidence that the pandemic also exacerbated these symptoms in females with FXS (62), the discussion that follows should be interpreted with the expectation of a parallel — and possibly amplified — post-pandemic increase in anxiety and depression within this population.

Although the meta-analysis estimates rates of any anxiety disorder, it is important to highlight that social anxiety is particularly prominent in females with FXS and typically intensifies around puberty (56, 67) — a developmental window that also marks the emergence of depressive symptoms (66, 67). Although this trajectory resembles population trends (median age of onset: 14 for social anxiety, 15 for depression; 104), anxiety in females with FXS often emerges far earlier. By preschool age, nearly one-third already meet diagnostic criteria for an anxiety disorder (49), and medication use has been reported as early as age five (55). Such early and persistent anxiety may become normalized or overlooked by caregivers and clinicians — a concerning possibility given that, in the general population, inadequately treated anxiety can increase risk for subsequent depression (105). Considering how prominent anxiety is in the clinical presentation of females with FXS, there is a compelling need for preventative interventions — such as parent-mediated early childhood programs (e.g., 106) — to mitigate symptom severity and reduce the likelihood of progression to co-occurring conditions like depression.

Depression itself represents a significant clinical concern, with an estimated prevalence of 41%. Despite this, depression remains comparatively understudied, with only about half as many published studies as those focused on anxiety. This discrepancy likely reflects a longstanding focus on male-driven psychiatric phenotypes in FXS, where anxiety has traditionally been viewed as the primary mental health concern (107). Consequently, much of the available evidence relies on broadband, informant-report measures such as the ADAMS (19), rather than structured diagnostic assessments. Advancing the field will require incorporating validated clinical tools aligned with diagnostic frameworks (e.g., DSM-5; 48) to generate more accurate prevalence estimates and clarify the presentation of depressive symptoms in females with FXS.

Developing a clearer understanding of how depression presents is also critical for distinguishing it from anxiety. Social withdrawal — a hallmark of the FXS behavioral phenotype — is a diagnostically recognized symptom of both conditions (48), yet within FXS it is often attributed primarily to anxiety (108). Clarifying when withdrawal signals emerging depression carries important clinical implications. Avoidant behavior in adolescence may progress into social isolation, contributing to the co-emergence of social anxiety and depression during puberty (66, 67). Moreover, affect problems predict reduced independence in adulthood for females with FXS (28). Without sensitive methods for differentiating anxiety from depression, interventions may be mistimed or misaligned, ultimately comprising long-term outcomes.

### 4.2 Sex-specific trends in ADHD

Meta-analytic findings indicate that ADHD affects approximately one-third (33%) of females with FXS – four times the prevalence observed in the general female population (8%; 109). Consistent with broader trends (110), ADHD in females with FXS is identified later than in males – 0% vs. 31% in early childhood (49) and 29% vs. 51% by adolescence (75). These disparities likely reflect differences in symptom presentation. The reviewed literature highlights that females with FXS typically show less hyperactivity but greater inattention symptoms than males (Baker et al., 2019; 12, 54, 71, 73, 76), mirroring sex-specific ADHD patterns in the general population (111). Because inattentive symptoms are more subtle, teachers and clinicians are less likely to identify ADHD in females with FXS (74) — a pattern also seen more broadly (112).

The delay and disparity in ADHD diagnosis among females can be traced, in part, to diagnostic frameworks developed predominantly from male-based samples (only 21% female; 113), which insufficiently capture the nuanced ways ADHD manifests in females. For females with FXS, this diagnostic gap may be further exacerbated by *diagnostic overshadowing*, whereby ADHD-related behaviors are minimized or attributed solely to the underlying genetic condition (41). More broadly, undiagnosed ADHD has well-documented effects on women’s emotional and social functioning (114) and is frequently misidentified as anxiety or depression (115). Conversely, symptoms of anxiety or mood disorders — such as distractibility, forgetfulness, and emotional reactivity — may themselves be mistaken for ADHD (116). Given the high prevalence of overlapping psychiatric features in females with FXS, ADHD should be understood within a comprehensive mental health framework rather than as an isolated diagnosis. Behaviors such as appearing disengaged in social interactions or preoccupied with worries or routines may, at times, reflect attentional regulation difficulties rather than anxiety or mood pathology — and vice versa.

### 4.3 Aggression and self-injury: secondary to co-occurring conditions?

Although aggression and self-injury are recognized characteristics of the FXS phenotype (18), they appear at relatively low rates in females (20% and 14%, respectively) relative to the other psychiatric symptoms discussed here. Notably, most prevalence estimates derive from studies focused primarily on males, in which females were included as comparison groups, rather than from research designed to investigate female-specific presentations. This differs from areas such as anxiety, depression, and ADHD, where there is some research focused exclusively on female presentations. Consequently, interpretations of the centrality of aggression and self-injury within the psychiatric profile of females with FXS should be approached with caution. Meta-regression analyses further support this view, showing that studies employing more comprehensive assessment tools and higher methodological quality reported substantially lower prevalence rates-approximately 10% for aggression and 5-7% for self-injury. This pattern suggests that less rigorous methodologies may overestimate the frequency of these behaviors in females with FXS. Moreover, higher rates of aggression and self-injury were observed in individuals with co-occurring anxiety or autism, implying that behaviors such as defiance, tantrums, arguing, or repetitive self-directed actions (e.g., rubbing, scratching) may reflect manifestations of these co-occurring conditions rather than core characteristics of FXS in females.

### 4.4 Autism and increased psychiatric vulnerability

Our review suggests that females with FXS who also meet criteria for autism may exhibit heightened psychiatric vulnerability, characterized by increased rates of anxiety, depression, aggression, and self-injury (56, 67, 69, 70). This co-occurrence likely reflects shared genetic liability, whereby overlapping genetic factors for autism and FXS jointly elevate baseline vulnerability. Behaviorally, symptoms and conditions often interact in reciprocal and reinforcing ways – for instance, autistic traits may intensify social withdrawal, which in turn heightens susceptibility to depression. The expression of autistic traits within the context of FXS may therefore create additional challenges in social, educational, and employment settings, introducing environmental stressors that amplify underlying genetic liability. Collectively, these interacting influences may manifest as greater psychiatric morbidity than would be expected in females with FXS alone. Accordingly, associations between autism and increased psychiatric risk in this group should be understood through the lens of shared genetic mechanisms and dynamic symptom interactions, rather than as straightforward one-directional causation.

Considering potential mechanistic pathways and symptom expression, social coping strategies may further contribute to psychiatric vulnerability. Among autistic females without FXS, the use of masking and compensatory behaviors – such as rehearsing conversations or maintaining forced eye contact – has been linked to heightened emotional exhaustion, depression, and suicidality (33, 35, 117, 118). Although not yet systematically examined in females with FXS, a similar conflict may exist given the tension between strong social motivation and pronounced social anxiety – a defining feature of the FXS phenotype (108). Such strategies may facilitate social engagement in the short term but often at the expense of long-term psychological well-being. Investigating whether females with FXS engage in comparable masking behaviors – and how these relate to mental health – represents an important avenue for future research, particularly given evidence from Hartley and colleagues (28) that, beyond affect problems, the ability to interact “appropriately” predicted greater adult independence among females with FXS.

Insights from this work could inform the adaptation of neurodiversity-affirming supports, such as social-skills and self-care programs designed for autistic females (119), to better meet the needs of females with FXS, who often receive autism-based interventions in the absence of FXS-specific options (120). Beyond its clinical relevance, this line of inquiry holds broader implications for understanding autism heterogeneity (121): as an X-linked condition, FXS offers a powerful model for investigating sex-specific mechanisms underlying autism presentation and psychiatric comorbidity.

### 4.5 Limitations and future directions

It is important to acknowledge that this review should not be regarded as an exhaustive account of psychiatric difficulties in females with FXS. Although the evidence presented highlights considerable psychiatric vulnerability in this group, the field has long been shaped by a disproportionate focus on males. As shown in this review, studies focused on psychiatric difficulties in females have been published at roughly one-quarter the rate of those focused on males. This disparity parallels broader NIH funding trends (122), wherein grants supporting research on males with FXS have outnumbered those including females. Given that the NIH is the largest source of research funding globally — and that U.S.-based studies constitute the majority of those included here (89%; *n* = 33) — there is a clear need to advocate for funding initiatives that encourage research specifically on females with FXS. Doing so is a crucial first step in addressing the methodological challenges required to capture the variability and develop a more nuanced understanding of this population.

Future research would benefit from incorporating comparisons with other neurodivergent female populations – an approach increasingly evident in recent studies (56, 60, 62, 65–67, 99). Such comparisons will be most valuable when neurodivergent females are considered not only as a reference group, but as a conceptual framework for shaping female-focused FXS research. This perspective would enable the systematic investigation of psychiatric difficulties frequently reported among neurodivergent females – such as panic attacks, obsessive-compulsive symptoms, eating disorders, and body dysmorphia (123) – within the context of FXS. Adopting this lens represents a meaningful shift away from traditional questions of whether females with FXS resemble affected males, toward a focus on defining their distinct experiences within the broader landscape of female neurodivergence.

Comparisons with males remain essential for evaluating the generalizability of clinical guidance in FXS and for identifying sex-specific patterns. However, a major limitation – both of this review and the broader literature – is the absence of psychiatric assessment tools validated across the full range of intellectual abilities observed in FXS. Even in studies that report IQ, the variability of the female phenotype appears underrepresented, with IQ data suggesting inclusion of predominantly females with borderline cognitive ability. It is also possible that the clinic-based samples reported here and included in meta-analyses – such as those by Visootsak and colleagues (75) – may overrepresent females with ID, given that they were recruited through specialist services; however, IQ data were not reported for that study.

The representativeness of samples is particularly important, as approximately one-third of females with FXS have ID (23), and evidence from adult populations indicates that cognitive level – beyond sex – plays a central role in shaping intervention needs. For example, psychological therapies such as mindfulness-based interventions and cognitive behavioral therapy (CBT) have demonstrated efficacy for anxiety, ADHD, social difficulties, and depression not only in females with FXS but also in males with mild ID (120). Future research should therefore aim to conduct sex-based comparisons within cognitively matched samples to better delineate subgroup-specific profiles (e.g., ID vs. non-ID, rather than male vs. female; 9). Achieving this will require more inclusive and representative sampling that captures the full cognitive and behavioral spectrum of FXS – with particular attention to female inclusion – supported by open-science practices and data sharing to more accurately reflect the breadth of psychiatric difficulties within this population.

Finally, study samples were strikingly homogeneous in geographic and demographic composition. Nearly all studies were conducted in the United States, with minimal representation from other regions, and the vast majority of participants were White and non-Hispanic. Socioeconomic data were rarely collected or insufficiently detailed to examine meaningful patterns. This lack of diversity substantially limits the generalizability of findings and leaves cultural, socioeconomic, and health system influences on the recognition and treatment of psychiatric difficulties in females with FXS poorly understood. Addressing these gaps will be essential for developing inclusive, equitable research frameworks and clinical practices for females with FXS.

### 4.6 Conclusions

Psychiatric difficulties – particularly anxiety, depression, and ADHD – are highly prevalent among females with FXS, challenging the persistent notion that they are only “mildly” affected. A persistent male bias in FXS research has likely obscured the scope and complexity of these challenges. Advancing the field requires greater attention to the timing, manifestation, and interplay of psychiatric symptoms within the broader context of neurodivergent female experiences. This emerging framework – supported by evidence linking co-occurring autism to heightened psychiatric vulnerability – reorients the focus from intellectual disability as the primary marker of clinical need in FXS toward recognizing mental health as a central to functional outcomes for females.

## Data Availability

The original contributions presented in the study are included in the article/**Supplementary Material**, further inquiries can be directed to the corresponding author/s.
