# Supplementary material for: Psychiatric difficulties in females with fragile X syndrome: a systematic review and meta-analyses
Source: Front Psychiatry. 2026 Jul 8;17:1886787. doi: 10.3389/fpsyt.2026.1886787 (PMC13390116; doi:10.3389/fpsyt.2026.1886787)
Supplement: Supplementary file 1 [file Table1.docx]

| Supplementary Table 1. Search Terms for Central Concepts of the Research Aims | |
| --- | --- |
| Fragile X | ("Fragile X" OR "Fragile X Syndrome" OR "Fragile-X" OR FXS OR "FRAXA Syndrome" OR AFRAX OR "Martin Bell* Syndrome" OR "Marker X Syndrome" OR "fraX Syndrome" OR "fra(X) Syndrome" OR "X-linked mental retardation" OR Macroorchidism OR "Escalante* Syndrome" OR Escalante* OR "FRAXE Syndrome" OR "Fragile X mental retardation" OR "Fragile X-F mental retardation" OR "Mar(X) Syndrome" OR "Mental retardation, X-linked" OR FMR1 OR FMRP) |
| Psychiatric terms | ("psychiatric disord*" OR "psychiatric prob*" OR "mental ill*" OR "mental disord*" OR psychopatholog* OR "emotional prob*" OR "emotional disord*" OR "behavioral prob" OR "behavioral disord" OR "psychological prob*" OR DSM* OR ICD* OR "psychiatrically disord*" OR psychosis OR psychotic OR anxi* OR depress* OR oppositional OR hyperactiv* OR ADHD OR "conduct disord*" OR obsess* OR phobi* OR "mood disord*" OR schizophren* OR bipolar OR anorex* OR bulimi* OR "challenging beh*" OR autis OR ASD OR "PDD-NOS" OR PDDNOS OR PDD OR Asperger* OR "pervasive developmental disorder*" |
| *Note.* the two lists were combined with the AND operator | |

Supplementary Table 2. Characteristics of Studies and Corresponding Meta-Analysis Results

|  | **Study** | **Quality rating** | **Age range** | **Assessment tool** | **Total N** | **Events** | **Effect size** | **95% confidence interval** | **Weight (random, %)** |
| --- | --- | --- | --- | --- | --- | --- | --- | --- | --- |
| **Anxiety** | Bailey et al. (2008) | 1.00 | Wide | Parent reported diagnosis | 259 | 145 | 0.24 | [-0.00, 0.49] | 22.10 |
|  | Wheeler et al. (2016) | 1.00 | Wide | Parent reported diagnosis | 119 | 63 | 0.12 | [-0.24, 0.48] | 20.60 |
|  | Cordeiro et al. (2011) | 2.33 | Wide | DSM-IV anxiety disorder | 39 | 30 | 1.20 | [0.46, 1.95] | 14.50 |
|  | Freund et al. (1993) | 2.67 | Wide | DICA-P | 17 | 11 | 0.61 | [-0.39, 1.60] | 11.10 |
|  | Muller et al. (2019) | 1.00 | Narrow | Qualitative interview with diagnosis and/or symptoms described by parent coded | 11 | 5 | -0.18 | [-1.37, 1.00] | 9.10 |
|  | Visootsak et al. (2016) | 1.67 | Wide | Medical records from specialist FXS clinic | 18 | 16 | 2.08 | [0.61, 3.55] | 6.80 |
|  | Roberts et al. (2020) | 2.67 | Narrow | PAPA | 14 | 4 | -0.92 | [-2.08, 0.24] | 9.30 |
|  | Wall et al. (2019) | 2.67 | Narrow | SCAS-P | 11 | 2 | -1.50 | [-3.04, 0.03] | 6.40 |
| **ADHD** | Freund et al. (1993) | 2.67 | Wide | DICA-P | 17 | 6 | -0.61 | [-1.60, 0.39] | 18.40 |
|  | Hagerman et al. (1992) | 2.33 | Wide | Conners Rating Scale (Conners, 1973) | 32 | 10 | -0.79 | [-1.54, -0.04] | 28.90 |
|  | Muller et al. (2019) | 1.00 | Narrow | Qualitative interview with diagnosis and/or symptoms described by parent coded | 11 | 7 | 0.56 | [-0.67, 1.79] | 12.80 |
|  | Reilly et al. (2015) | 1.00 | Wide | Parent reported diagnosis | 21 | 5 | -1.16 | [-2.17, -0.16] | 18.10 |
|  | Sullivan et al. (2006) | 1.33 | Narrow | CSI-PC; ASI-PC; CSI-TC; ASI-TC; CBCL | 6 | 1 | -1.61 | [-3.76, 0.54] | 4.50 |
|  | Visootsak et al. (2016) | 1.67 | Wide | Medical records from specialist FXS clinic | 18 | 5 | -0.96 | [-1.99, 0.08] | 17.30 |
| **Depression** | Hartley et al. (2011) | 1.00 | Wide | Parent reported diagnosis | 89 | 53 | 0.39 | [-0.04, 0.81] | 26.40 |
|  | Hessl et al. (2001) | 2.33 | Wide | CBCL | 40 | 16 | -0.41 | [-1.04, 0.23] | 24.80 |
|  | Freund et al. (1993) | 2.67 | Wide | DICA-P | 17 | 8 | -0.12 | [-1.07, 0.83] | 21.60 |
|  | Bailey et al. (2008) | 1.00 | Wide | Parent reported diagnosis | 259 | 57 | -1.27 | [-1.56, -0.97] | 27.20 |
| **Aggression** | Arpone et al. (2022) | 2.33 | Wide | ABC | 21 | 10 | -0.10 | [-0.95, 0.76] | 16.60 |
|  | Muller et al. (2019) | 1.00 | Wide | Qualitative interview with diagnosis and/or symptoms described by parent coded | 11 | 4 | -0.56 | [-1.79, 0.67] | 11.80 |
|  | Visootsak et al. (2016) | 1.67 | Wide | Medical records from specialist FXS clinic | 18 | 0 | -3.56 | [-6.37, -0.74] | 3.50 |
|  | Hartley et al. (2011) | 1.00 | Wide | Parent reported diagnosis | 89 | 12 | -1.86 | [-2.47, -1.25] | 20.60 |
|  | Bailey et al. (2008) | 1.00 | Wide | Parent reported diagnosis | 259 | 36 | -1.82 | [-2.18, -1.47] | 24.60 |
|  | Wheeler et al. (2016) | 1.00 | Wide | Parent reported diagnosis | 119 | 21 | -1.54 | [-2.01, -1.07] | 22.90 |
| **Self-injury** | Hall et al. (2008) | 2.67 | Wide | SIB-C | 29 | 5 | -1.57 | [-2.53, -0.61] | 10.50 |
|  | Muller et al. (2019) | 1.00 | Wide | Qualitative interview with diagnosis and/or symptoms described by parent coded | 11 | 1 | -2.30 | [-4.36, -0.25] | 2.50 |
|  | Symons et al. (2010) | 1.33 | Wide | Parent report (survey) | 51 | 9 | -1.54 | [-2.26, -0.82] | 17.60 |
|  | Hartley et al. (2011) | 1.00 | Wide | Parent reported diagnosis | 89 | 15 | -1.60 | [-2.15, -1.04] | 27.00 |
|  | Bailey et al. (2008) | 1.00 | Wide | Parent reported diagnosis | 259 | 2 | -2.19 | [-2.60, -1.79] | 42.50 |

*Note.* The assessment tool indicates the diagnostic or reporting instrument used. Study quality ratings are based on quality/risk of bias criteria (Marlborough et al., 2021; Richards et al., 2015), with higher scores reflecting stronger quality. Age range was coded as narrow when limited to a single developmental stage (e.g., preschool only) and as wide when spanning multiple stages (e.g., preschool through adulthood). Effect sizes are presented as log odds ratios (prior to back-transformation, as reported in the paper). Study weights are derived from the random-effects model.

Supplementary Table 3. Pooled Random-Effects Estimates and Heterogeneity Statistics by Psychiatric Difficulty

|  | Effect size | 95% confidence interval | *p*-value | Heterogeneity |
| --- | --- | --- | --- | --- |
| **Anxiety** | 0.26 | [-0.19, 0.72] | .259 | *I²* = 69.2%, *τ^2^* = .228, *p* = .002 |
| **ADHD** | -0.72 | [-1.18, -0.25] | .003 | *I²* = 14.6%, *τ^2^* = .050, *p* = .320 |
| **Depression** | -0.37 | [-1.30, -0.56] | .439 | *I²* = 92.8%, *τ^2^* = .808, *p* < .001 |
| **Aggression** | -1.39 | [-1.95, -0.83] | .001 | *I²* = 73.4%, *τ^2^* = .299, *p* = .002 |
| **Self-injury** | -1.85 | [-2.18, -1.53] | .001 | *I²* = 15.1%, *τ^2^* = .022, *p* = .318 |

*Note.* Pooled effect sizes represent the results of random-effects, inverse-variance meta-analyses of logit-transformed proportions for each psychiatric difficulty. Heterogeneity statistics are provided, with I² indicating the proportion of variance due to heterogeneity, τ² the estimated between-study variance, and *p* the significance test of heterogeneity.
